# Meteorological Register for May, 1817

**Published:** 1817-07

**Authors:** John Bailey

**Affiliations:** Surgeon, &c.


### Meteorological Register for May, 1817,

*Kept at Harwich, in Essex,*

By JOHN BAILEY, Surgeon, &c.

| Moon's Age. | | Atmospherical Preſſure. Morn. | Ev. | Temp. | Wind. | Remarks. | General Statement of Diſeaſes. |
|---|---|---|---|---|---|---|---|
| ● | 1 | 29 7 | 29 7 | 48 | NE | Rain. Windy | |
| | 2 | 8½ | 8½ | 51 | NE | ditto | |
| | 3 | 8 | 7 | 59 | | Cloudy | Pneumonia |
| | 4 | 8 | 8 | 61 | NW | ditto | |
| | 5 | 30 | 30 | 60 | NE | Fine and clear | |
| | 6 | 1 | 1 | 61 | SE | ditto | Catarrhus |
| | 7 | 2 | 29 3½ | 56 | E | ditto | |
| ☽ | 8 | 29 8 | 7½ | 50 | E | Rain | Intermittens |
| | 9 | 7½ | 6 | 50 | NE | Brisk Gale | |
| | 10 | 5½ | 5½ | 56 | SE | Rain | Varicella |
| | 11 | 4½ | 4 | 55 | W | ditto | |
| | 12 | 3 | 4 | 57 | W | ditto | Parotidea |
| | 13 | 6 | 7 | 58 | W | ditto | |
| | 14 | 6 | 5 | 56 | SW | Rn. Hail storm | |
| | 15 | 5 | 6 | 56 | SW | Fine | |
| ○ | 16 | 6 | 6 | 58 | SW | ditto | |
| | 17 | 7 | 6 | 58 | SW | | |
| | 18 | 4 | 4 | 59 | S | Rain | |
| | 19 | 3 | 4 | 58 | NE | ditto | |
| | 20 | 4½ | 4½ | 59 | E | ditto | |
| | 21 | 3½ | 4 | 51 | SW | ditto | |
| | 22 | 4 | 4½ | 56 | S | ditto | |
| | 23 | 4½ | 4½ | 58 | S | | |
| ☾ | 24 | 5 | 5 | 60 | SW | | |
| | 25 | 3½ | 2½ | 56 | E | Rain | |
| | 26 | 2½ | 3 | 56 | E | | |
| | 27 | 4 | 5 | 59 | S | | |
| | 28 | 6 | 6 | 57 | NW | Rain | |
| | 29 | 6 | 6½ | 52 | N | Much rain & wind | |
| ● | 30 | 7 | 7 | 53 | N | Brisk Gale | |
| | 31 | 8 | 8 | 54 | N | Rain. Ditto | |

Cold winds, with rain, retarding the progress of vegetation, generally, in the spring season of the year, produce intermittent forms of disease, combined, for the most part, with some affection of the breast. In debile habits, and amongst a certain class of persons, where poverty circumscribes both the means of cure and of comfort, an almost unmanageable disorder arises: we have seen this happen at every Equinox for many years past. The present has produced less instances of this sort than former ones.—An epidemic, resembling a very mild species of rubeola, has prevailed much among young children: a slight eruption has been visible; the febrile symptoms have run high, and the affection in the chest has been severe, several very young children having died. Varicella and parotidea have likewise been frequent; the latter, which usually is so mild as not to demand medical interference, has been very violent, and of considerable duration.

